# Supplementary material for: Efficient and safe therapeutic use of paired Cas9-nickases for primary hyperoxaluria type 1
Source: EMBO Mol Med. 2024 Jan 5;16(1):8. doi: 10.1038/s44321-023-00008-8 (PMC10897483; doi:10.1038/s44321-023-00008-8)
Supplement: Supplementary file 1 — Appendix [file 44321_2023_8_MOESM1_ESM.pdf]

Appendix

Supplemental Data

Efficient and Safe Therapeutic Use of Paired Cas9-Nickases for Primary Hyperoxaluria Type 1

Table of Contents

Appendix Figure S1 – Comparison of nuclease SaCas9 and paired SaCas9 nickases efficiency in transfected HEK293T cells (page 2).

Appendix Figure S2 – Paired D10ASaCas9 results in highly efficient gene disruption *in vivo* (page 3).

Appendix Figure S3 – Paired D10ASaCas9 results in heterogeneous modification on-target and a low AAV integration rate (page 4).

Appendix Figure S4 – Paired wtSaCas9 results in controlled editing on-target and a higher frequency of AAV integration (page 5).

Appendix Figure S5 – wtSaCas9-sgRNA-editing mainly results in small modifications on-target (page 6).

Appendix Figure S6 – Characterization of wtSaCas9-sgRNA-editing and targeted AAV integration (page 7).

Appendix Figure S7 – All-in-one AAV-D10ASaCas9 results in efficient gene disruption at each of the 3 tested doses (page 8)**.**

Appendix Figure S8 – All-in-one AAV-D10ASaCas9 results in highly efficient on-target editing and further reduced AAV integration rate (page 9).

Appendix Figure S9 – Comparison of TBG-D10A-g1+g2 and AAT-D10A-g1+g2 transduction efficiency *in vivo*, at the same tested dose (page 10).

Appendix Figure S10 – Comprehensive CAST-Seq analysis demonstrate the specificity of the selected gRNAs (page 11).

Appendix Table S1 – List of primers and gRNAs used in this study (page 12).

Appendix Table S2 – List of antibodies used in this study (page 13).

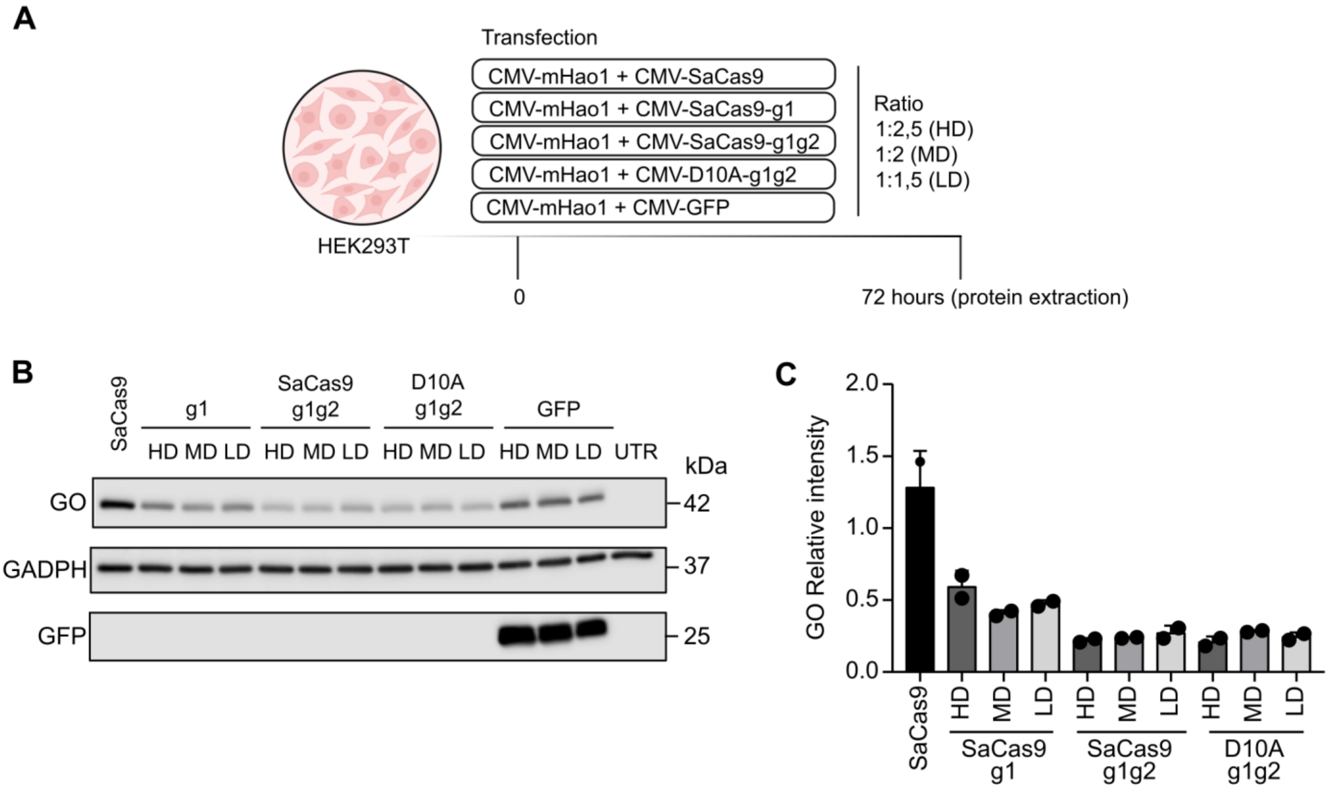

Appendix Figure S1 – Comparison of nuclease SaCas9 and paired SaCas9 nickases efficiency in transfected HEK293T cells.

A Schematic representation of the transfection performed in HEK293T cells. Five mixes of two plasmids per mix were transfected, at three different ratios (CMV-Hao1 + CMV-SaCas9; CMV-Hao1 + CMV-SaCas9-g1; CMV-Hao1 + CMV-SaCas9-g1g2; CMV-Hao1 + CMV-D10Ag1g2; CMV-Hao1 + CMV-GFP; at 1:2,5 (High Dose, HD); 1:2 (Medium Dose, MD); 1:1.5 (Low Dose, LD) each). Untransfected (UTR) cells were included as negative control.

B WB analysis of GO and GFP protein levels. GAPDH was used as a loading control.

C Relative quantification of GO bands density by WB, normalized to the respective GAPDH.

Data information: In (**A-C**) n = 2 technical replicate for each condition. In (**B**) n = 1 biological replicate per condition is shown. In (**C**) data are presented as mean ± SD.

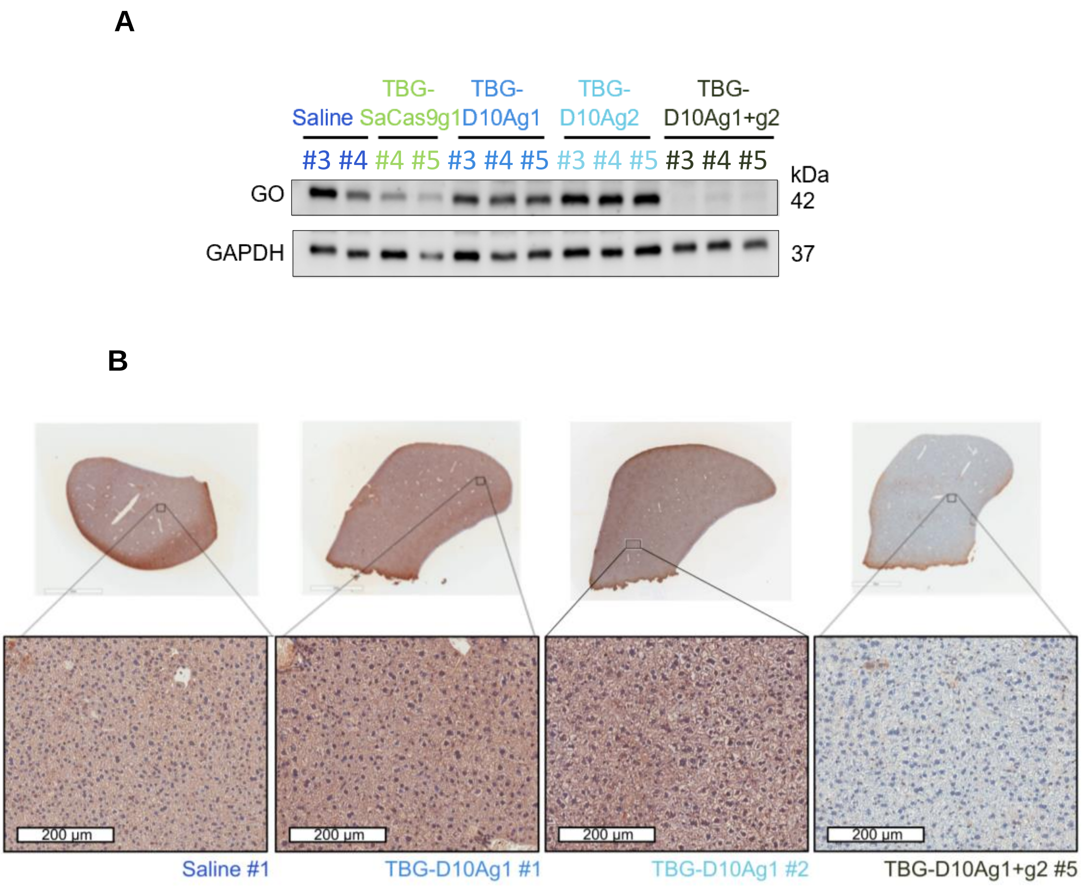

Appendix Figure S2 – Paired D10ASaCas9 results in highly efficient gene disruption *in vivo*.

A WB analysis of GO protein levels of PH1 animals injected with saline (n = 2), TBG SaCas9g1 (n = 2), TBG-D10Ag1 (n = 3), TBG-D10Ag2 (n = 3), not shown in main Fig. 1C and TBG-D10Ag1+g2 (n = 3). GAPDH was used as a loading control.

B 20X magnification of the specific sections of liver lobes represented in Fig 1D. Scale bar: 200 µm.

A

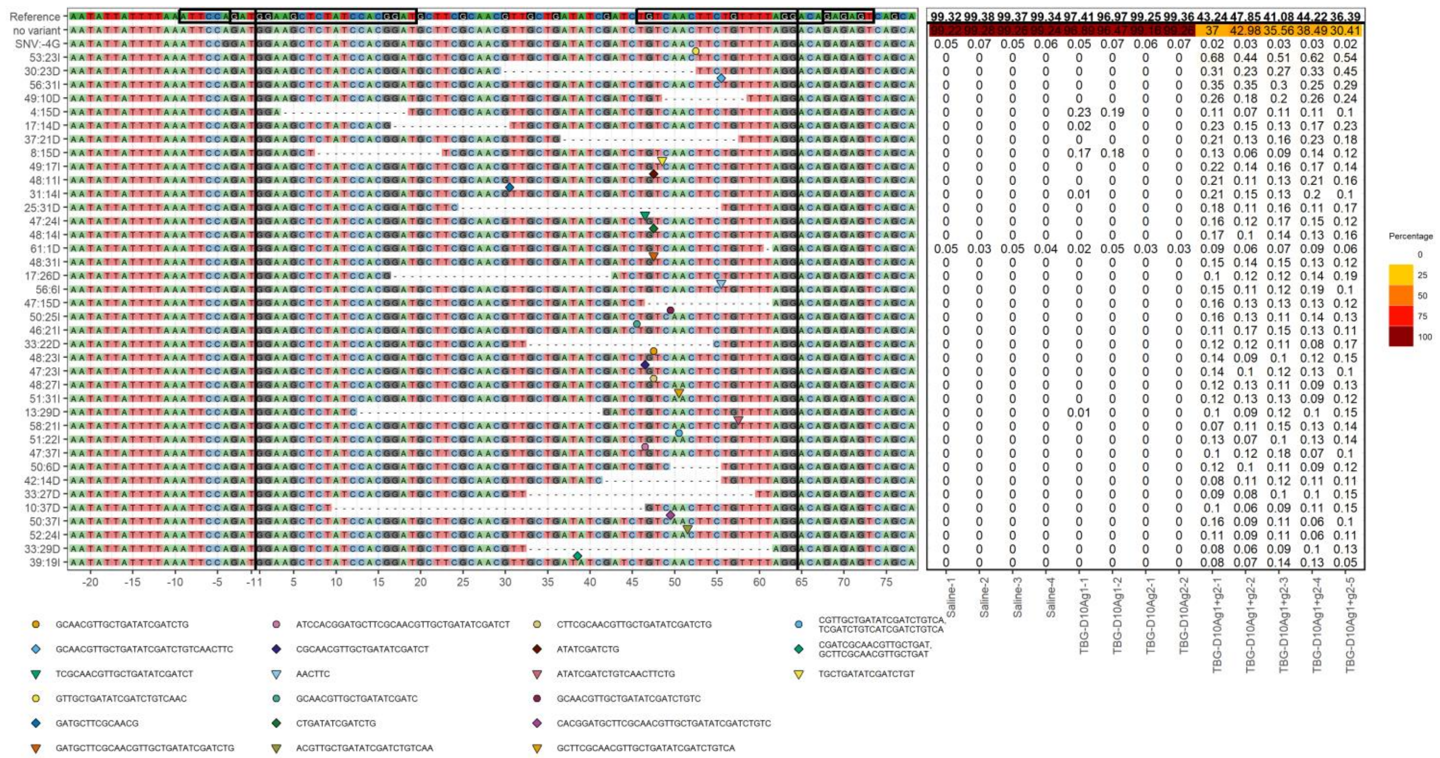

B

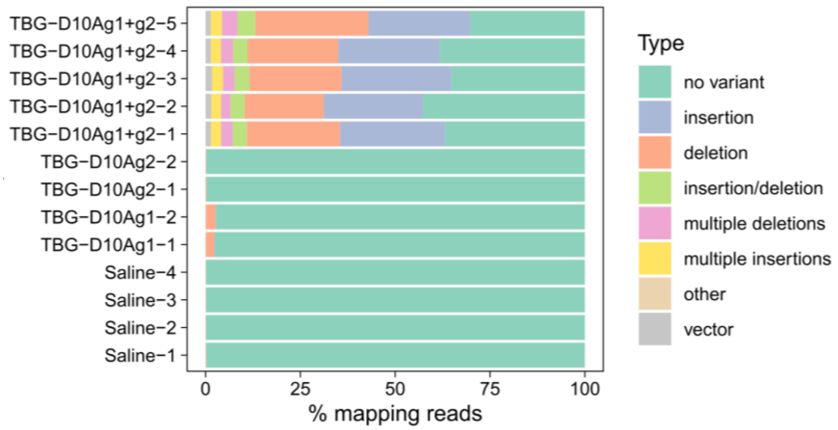

Appendix Figure S3 – Paired D10ASaCas9 results in heterogeneous modification on-target and a low AAV integration rate.

A Summary of the most frequent variant types, locations, and frequencies across multiple PH1 mice injected with saline (n = 4); TBG-D10Ag1 (n = 2); TBG-D10Ag2 (n = 2); TBG-D10Ag1+g2 (n = 5). The black blocks represent the target site and PAM sequence, while the black lines are the expected cut site. The size and location of the indel are described on the left. Deletions are represented with dashed lines, while insertions are with colored symbols. The sequences of the insertions are given at the bottom and showed 100% identity with the targeted locus. Representative indel variants are plotted according to frequency.

B Bar chart with the frequency of alleles variants (Saline (n = 4); TBG-D10Ag1 (n = 2); TBG-D10Ag2 (n = 2); TBG-D10Ag1+g2 (n = 5).

A

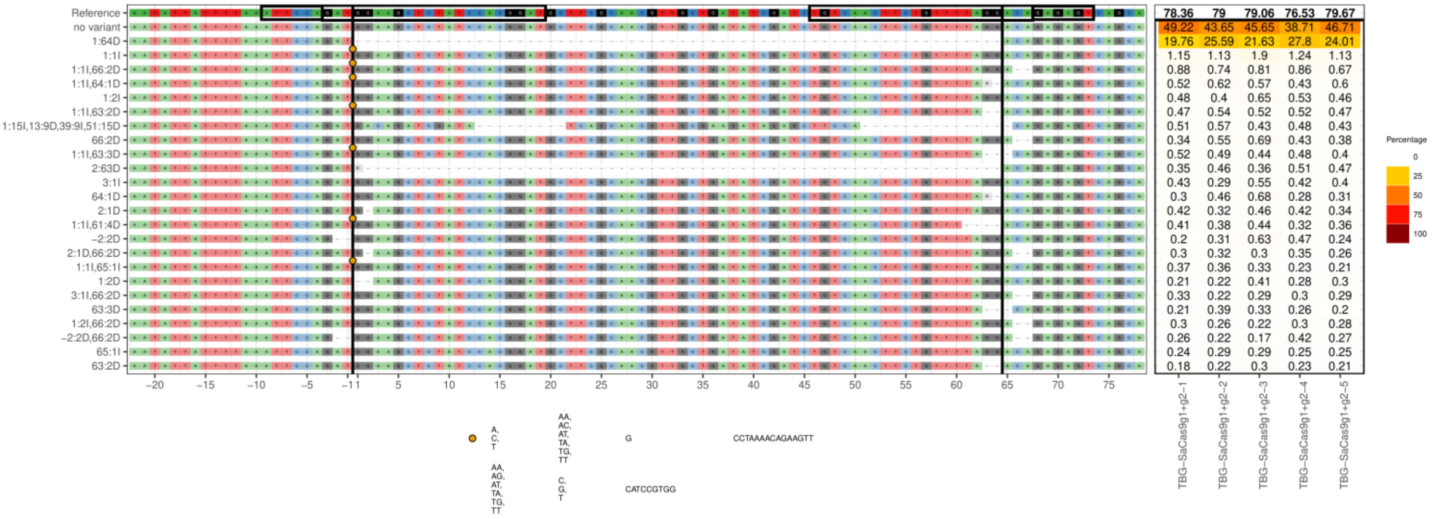

B

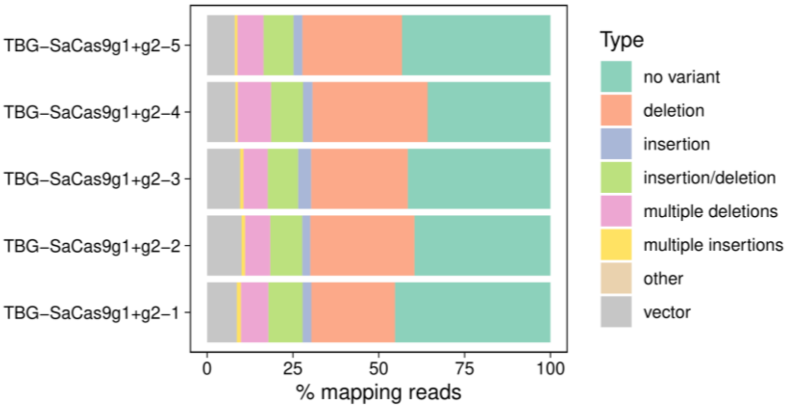

Appendix Figure S4 – Paired wtSaCas9 results in controlled editing on-target and a higher frequency of AAV integration.

A Summary of the most frequent variant types, locations, and frequency across multiple PH1 mice injected with TBG-SaCas9g1+g2 (n = 5). The black blocks represent the target site and PAM sequence, while the black lines are the expected cut site. The size and location of the indels are described on the left. Deletions are represented with dashed lines, while insertions are with colored symbols. The sequence of the insertion is described at the bottom. Representative indel variants are plotted according to frequency.

B Bar chart with the frequency of allele variants (TBG-SaCas9g1+g2 (n = 5)).

A

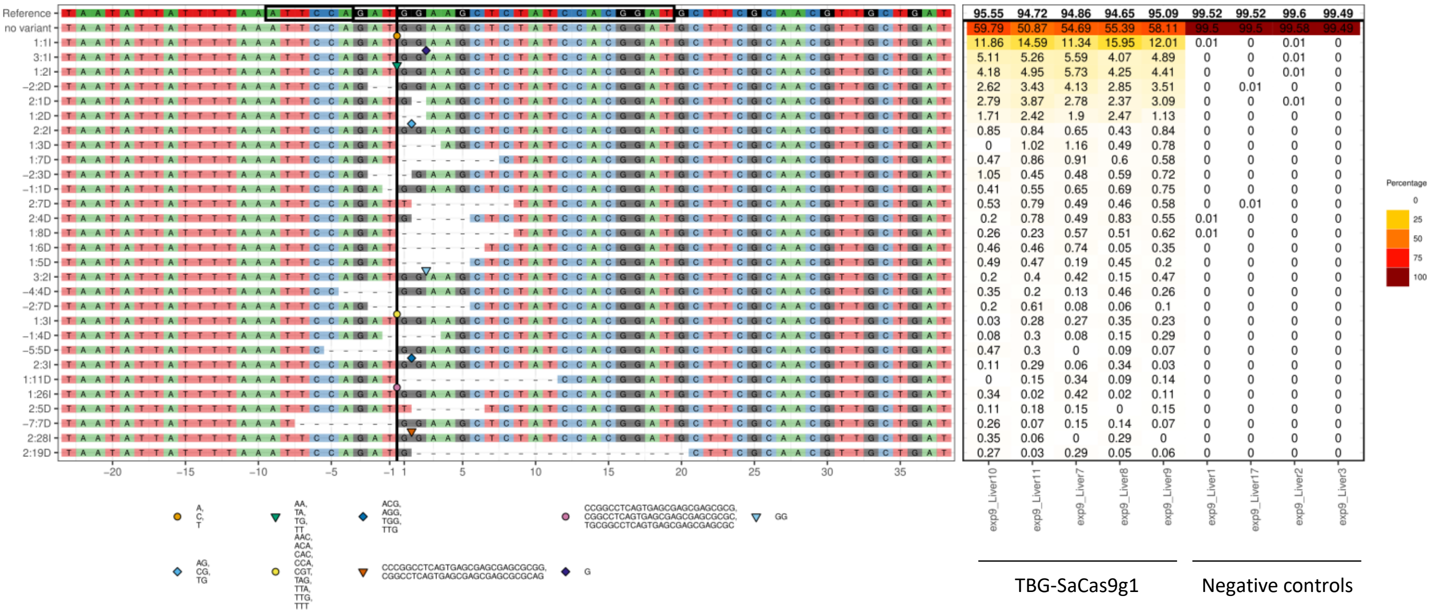

B

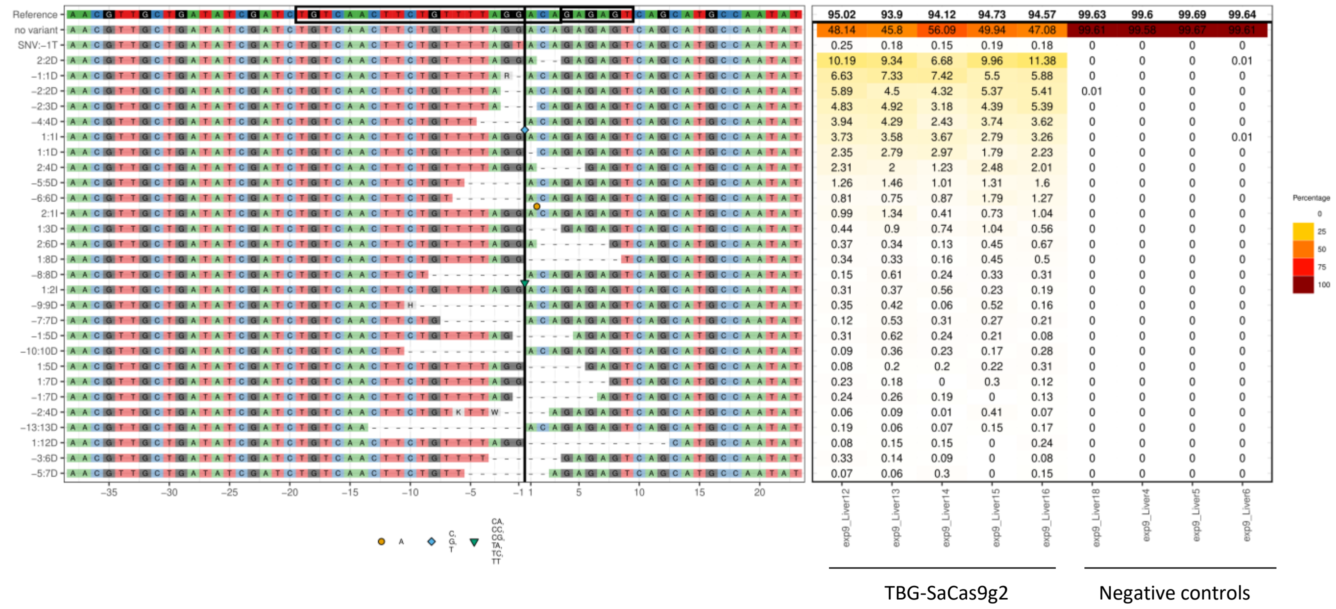

Appendix Figure S5 – wtSaCas9-sgRNA-editing mainly results in small modifications on-target.

A, B Summary of the most frequent variant types, locations, and frequency across multiple PH1 mice injected with TBG-SaCas9g1 (n = 5 exp_9Liver7-11), and negative controls (TBG-SaCas9 (n = 3 exp_9Liver1-3), saline solution (n = 1 exp_9Liver17)) (**A**); TBG-SaCas9g2 (n = 5 exp_9Liver12-16), and negative controls (TBG-SaCas9 (n = 3 exp_9Liver4-6), Saline solution (n = 1 exp_9Liver18)) (**B**). The black blocks represent the target site and PAM sequence, while the black lines are the expected cut site. The size and location of the indels are given on the left. Deletions are represented with dashed lines, while insertions are with colored symbols. The sequences of the insertions are given at the bottom. Representative indel variants are plotted according to frequency.

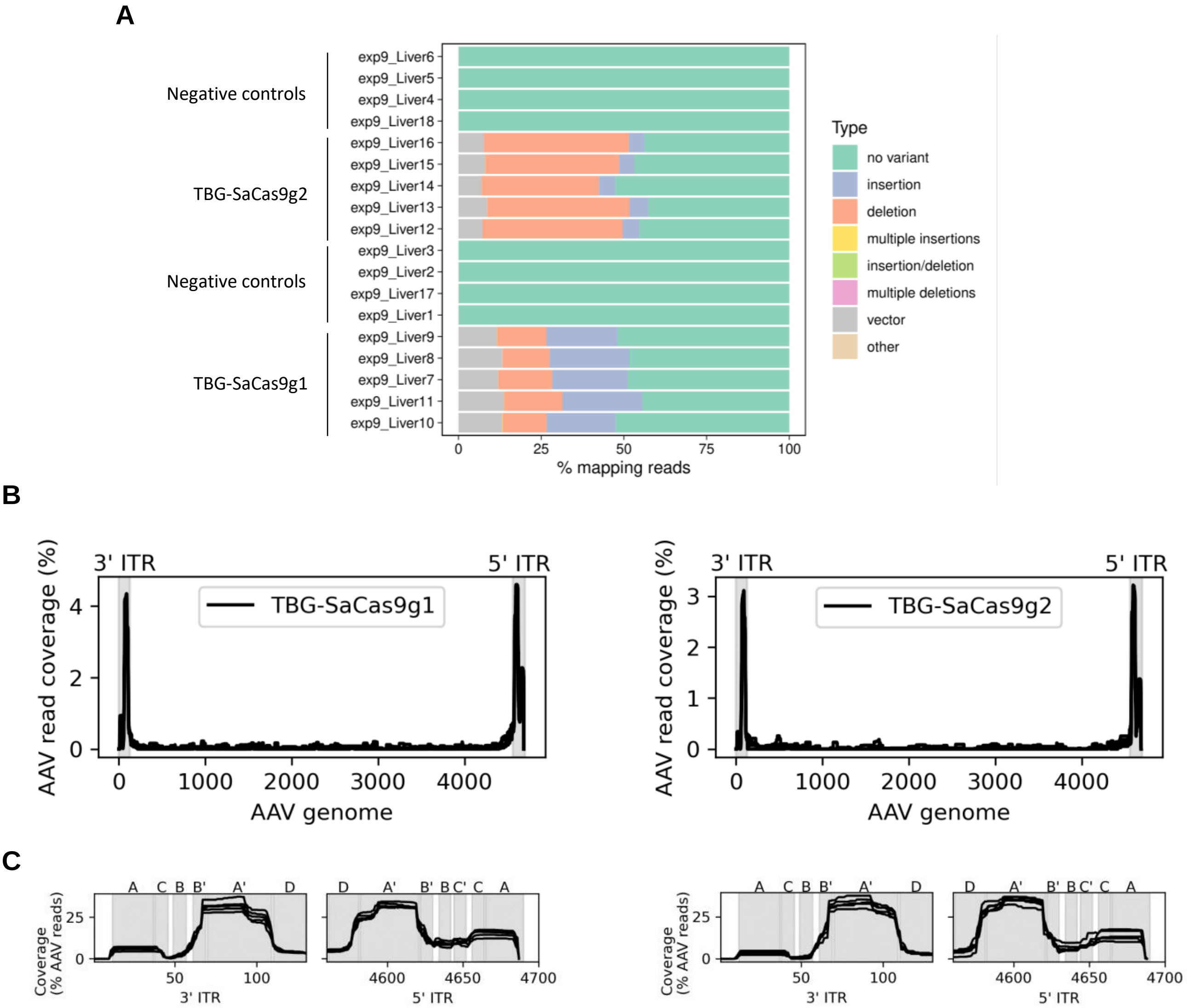

Appendix Figure S6 – Characterization of wtSaCas9-sgRNA-editing and targeted AAV integration.

A Bar chart with the frequency of allele variants of PH1 mice injected with TBG-SaCas9g1 (n = 5 exp_9Liver7-11), TBG-SaCas9g2 (n = 5 exp_9Liver12-16), and negative controls (TBG-SaCas9 (n = 6 exp_9Liver1-6), saline solution (n = 2 exp_9Liver17-18)).

B Coverage of integrated sequences across the AAV genome sequence (TBG-SaCas9g1 on the left and TBG-SaCas9g2 on the right). 50 bp windows along the AAV genome were used to represent the AAV read count.

C Coverage of 5’ and 3’ ITR integrated regions in the targeted locus (TBG-SaCas9g1 on the left and TBG-SaCas9g2 on the right). The drop in coverage demonstrates a preferred breakpoint between the B′–B and A′–A-arms.

A

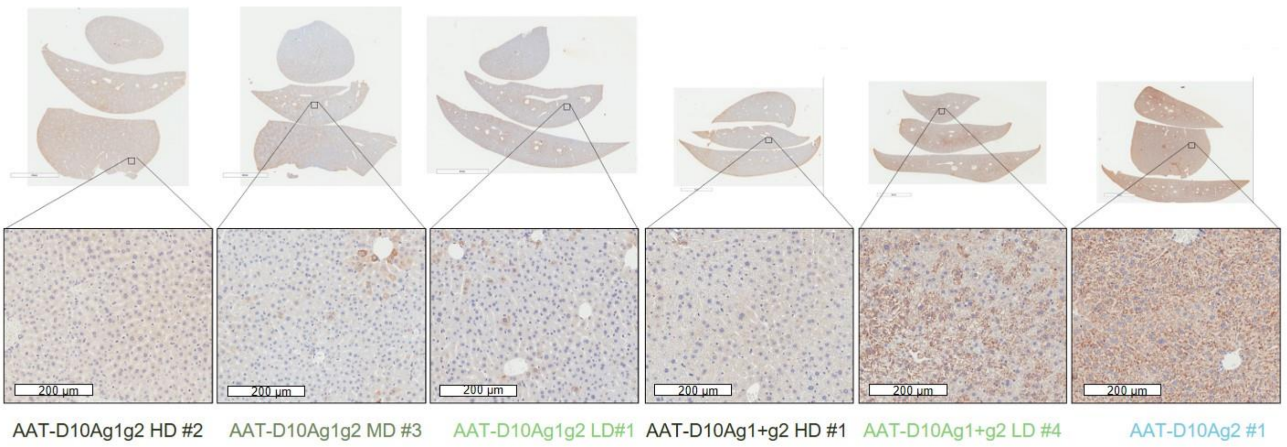

B

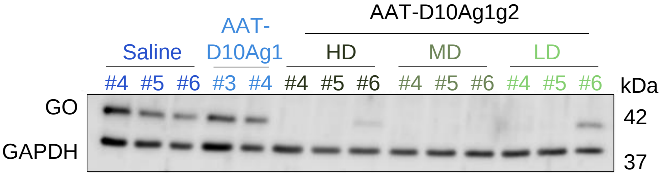

Appendix Figure S7 – All-in-one AAV-D10ASaCas9 results in efficient gene disruption at each of the 3 tested doses.

A 20X magnification of the specific sections of the liver lobes that are presented in main Fig 3B. Scale bar: 200 µm.

B WB of GO protein levels of PH1 animals injected with saline solution (Saline, n = 3), AAT-D10Ag1 (n = 2); AAT-D10Ag1g2 HD (n = 3); AAT-D10Ag1g2 MD (n = 3); AAT-D10Ag1g2 LD (n = 3) not shown in Fig 3C. GAPDH was used as a loading control.

A

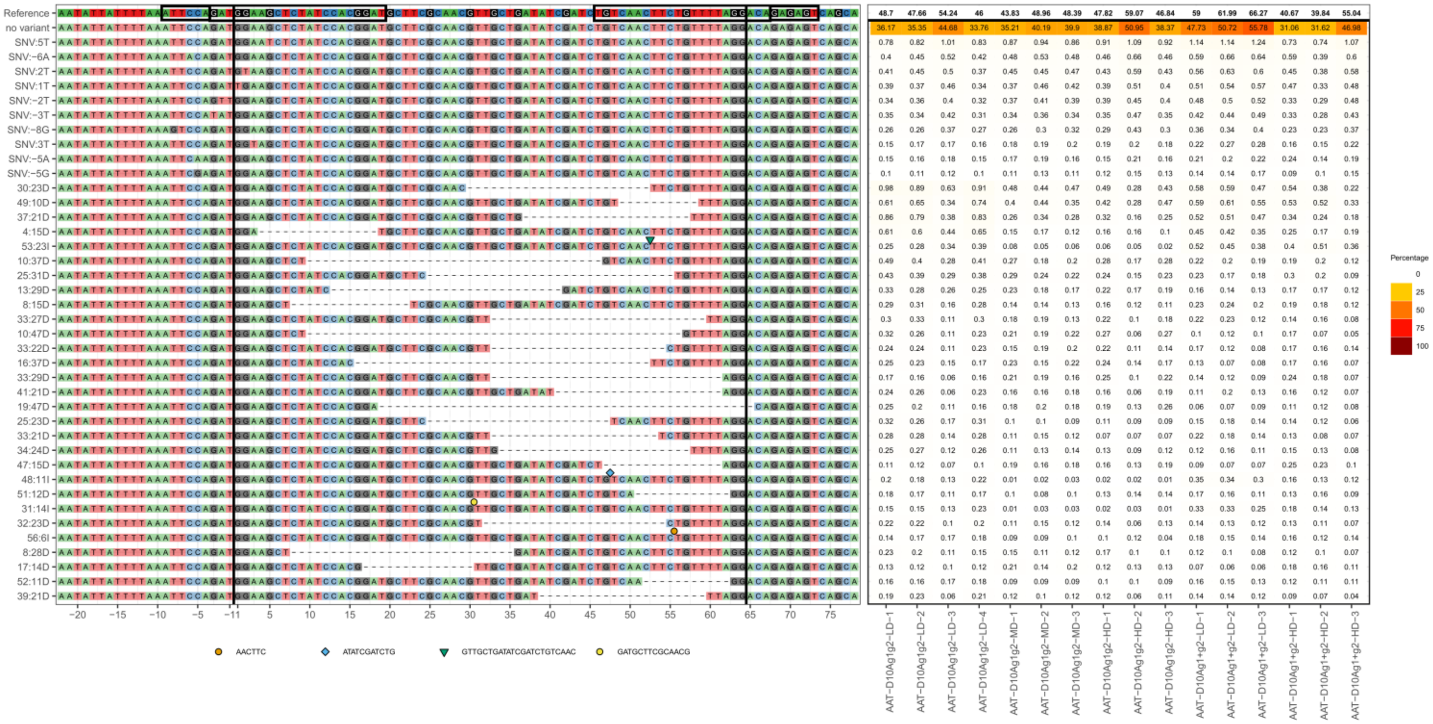

B

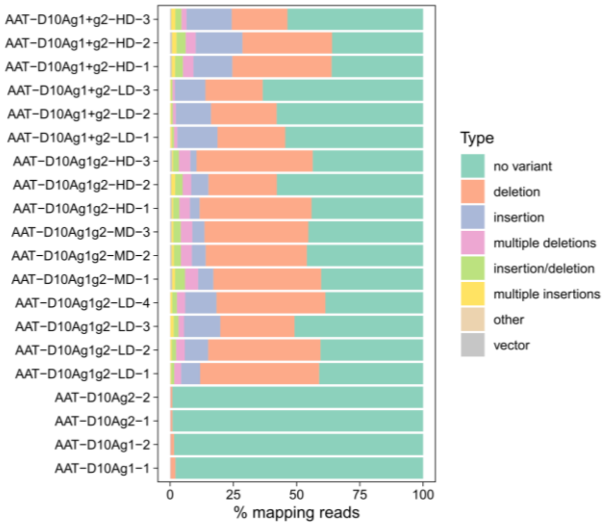

Appendix Figure S8 – All-in-one AAV-D10ASaCas9 results in highly efficient on-target editing and further reduced AAV integration rate.

A Summary of most frequent variant types, locations, and frequency across multiple PH1 injected with AAT-D10Ag1 (n = 2); AAT-D10Ag2 (n = 2); AAT-D10Ag1+g2 LD (n = 3); AAT-D10Ag1+g2 HD (n = 3); AAT-D10Ag1g2 LD (n = 4); AAT-D10Ag1g2 MD (n = 3); AAT-D10Ag1g2 HD (n = 3). The black blocks represent the target site and PAM sequence, while the black lines are the expected cut site. The size and location of the indels are given on the left. Deletions are represented with dashed lines, while insertions are with colored symbols. The sequence of the insertion is described at the bottom and showed 100% identity with the targeted locus. Representative indel variants are plotted according to frequency.

B Bar chart with the frequency of allele variants measured in PH1 mice injected with AAT-D10Ag1 (n = 2); AAT-D10Ag2 (n = 2); AAT-D10Ag1+g2 LD (n = 3); AAT-D10Ag1+g2 HD (n = 3); AAT-D10Ag1g2 LD (n = 4); AAT-D10Ag1g2 MD (n = 3); AAT-D10Ag1g2 HD (n = 3).

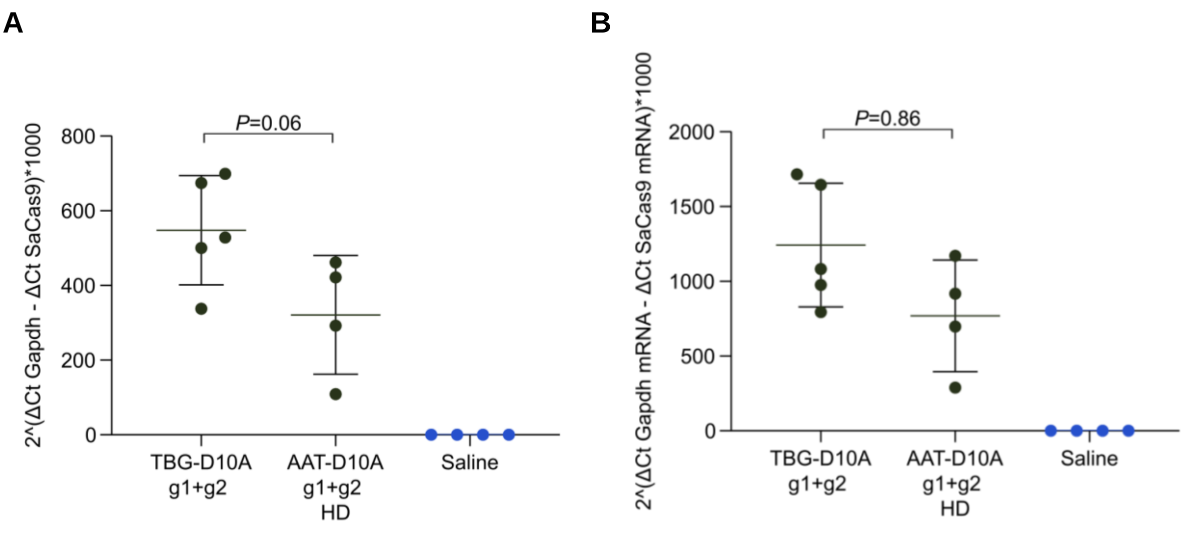

Appendix Figure S9 – Comparison of TBG-D10A-g1+g2 and AAT-D10A-g1+g2 transduction efficiency *in vivo*, at the same tested dose.

A Relative quantification of viral genome copies in the liver of treated and control animals.

B Relative expression of D10ASaCas9 in the liver of treated and control animals.

Data information: In (**A-B**) data are presented as mean ± SD (Unpaired two-tailed t-test, *ns*); n ≥ 4 mice per group.

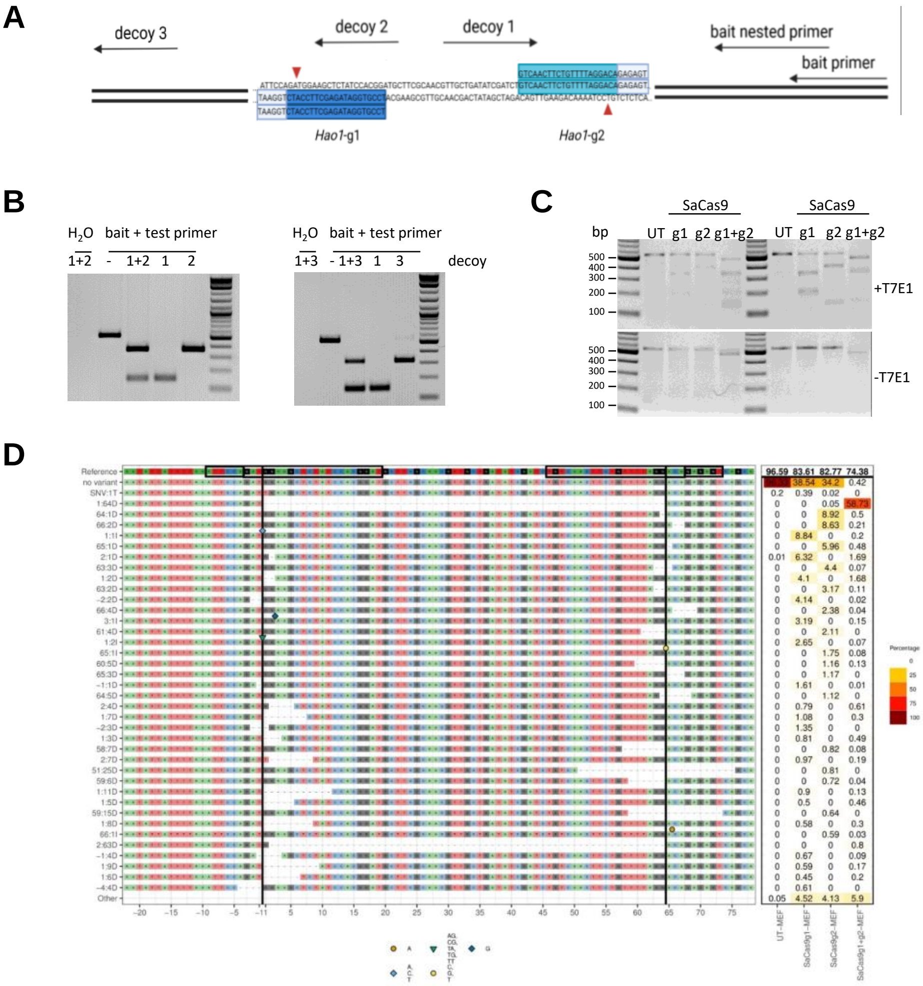

Appendix Figure S10 – Comprehensive CAST-Seq analysis demonstrate the specificity of the selected gRNAs.

A Scheme of decoy and bait primer test system.

B PCR validation of decoy primer efficiency. The reactions were resolved in a 2% agarose gel.

C T7E1 performed in MEF UT (n = 2) and treated with SaCas9 RNP+g1 (n = 2); SaCas9 RNP+g2 (n = 2); SaCas9 RNP g1+g2 (n = 2).

D Summary of most frequent variant types, locations, and frequency in UT MEF (n = 1) and MEF nucleofected with SaCas9 RNP+g1 (n = 1); SaCas9 RNP+g2 (n = 1); SaCas9 RNP g1+g2 (n = 1). The black blocks represent the target site and PAM sequence, while the black lines are the expected cut site. The size and location of the indels are given on the left. Deletions are represented with dashed lines, while insertions are with colored symbols. The sequence of the insertions is described at the bottom. Representative indel variants are plotted according to frequency.

Appendix Table S1. List of primers and gRNAs used in this study

Primer name	Sequence	Method
nSaCas9 D10A Fw	5'-GGTGATGCCGATGGCCAGGCCCAGGAT-3'	Mutagenesis
nSaCas9 D10A Rv	5'-ATCCTGGGCCTGGCCATCGGCATCACC-3'	
NheI-Hao1 CDS Fw	5'-attaaGCTAGCATGTTGCCTCGACTGGTCTGC-3'	Hao1 plasmid cloning
NotI-Hao1 CDS Rv	5'-gaactGCGGCCGCTCAGATCTTGGAAACAGCCA-3'	
NotI-Hao1 3UTR Fw	5'-taaaGCGGCCGCCAGTGCACAATATTTTCC-3'	
NotI-Hao1 3UTR Rv	5'-aactGCGGCCGCTGTGATAAAAGGAAAGAC-3'	
SaCas9 Fw	5'- AAGCCATCCCTCTGGAAGAT-3'	Viral genome copies quantification and SaCas9 expression
SaCas9 Rv	5'-TGCCCTTCTTGCTGTTTTCT-3'	
Gapdh Fw	5'-TGCACCACCAACTGCTTA-3'	
Gapdh Rv	5'-GGATGCAGGGATGATGTTC-3'	
ITR Fw	5'-GGAACCCCTAGTGATGGAGTT-3'	AAV Titration
ITR Rv	5'-CGGCCTCAGTGAGCGA-3'	
Hao1-int1 Fw	5'-CCAAAGCCTATAAGGGGATG-3'	On-target PCR and NGS (mice)
Hao1-int2 Rv	5'-CATCCTAGGAAGGGTGTTCG-3'	
Hao1-ex2 Fw	5'-ACACTCTTTCCCTACACGACGCTCTTCCGATCTagaccaatgtttgtcagagg-3'	
Hao1-ex2 Rv	5'-GACTGGAGTTCAGACGTGTGCTCTTCCGATCTtaaaagcatcctaggaaggg-3'	
7783 Fw	5'-CAGACCAATGTTTGTCAGAGG-3'	On-target PCR and NGS (MEF)
7784 Rv	5'-CATCCTAGGAAGGGTGTTCG-3'	
6878 Fw	5'-TCCAGGAGGTGAAATGGATC-3'	On-target PCR T7E1 (MEF)
Hao1-int2 Rv	5'-CATCCTAGGAAGGGTGTTCG-3'	
7659 Fw 1	5'-GAGTCTTGTGTCCCAGTTACCAGGcgattctgtaacactacttcggac-3'	Long Read Seq. Chr.8 MEF-UT
7663 Rv1	5'-CGGATACTAAGTTATCCTGCTGCGtgagtatcggcttcatgagacc-3'	
7660 Fw2	5'-TTCGGATTCTATCGTGTTTCCCTAcgattctgtaacactacttcggac-3'	Long Read Seq. Chr.8 MEF-g1
7664 Rv2	5'-TCATCGGTTAACGTACATCTGCGCtgagtatcggcttcatgagacc-3'	
7661 Fw3	5'-CTTGTCCAGGGTTTGTGTAACCTTcgattctgtaacactacttcggac-3'	Long Read Seq. Chr.8 MEF-g2
7665 Rv3	5'-ACTCGCTTTCTAAAGTTGACACACtgagtatcggcttcatgagacc-3'	
7662 Fw4	5'-TTCTCGCAAAGGCAGAAAGTAGTCcgattctgtaacactacttcggac-3'	Long Read Seq. Chr.8 mice
7666 Rv4	5'-GGCTCATATGTAACAAGCAGTAGGtgagtatcggcttcatgagacc-3'	
4032 linker prey	5'-GTAATACGACTCACTATAGGGC-3'	CAST-Seq
4033 nested linker prey	5'-ACACTCTACACTCTTTCCCTACACGACGCTCTTCCGATCTAGGGCTCCGCTTAAGGGAC-3'	
6871 bait	5'-CTCTTTGTCTCTCTTTTCTTACCTC-3'	
6873 decoy1	5'-CGTTGCTGATATCGATCTGTC-3'	
6875 decoy 2	5'-ATCCGTGGATAGAGCTTCC-3'	
6916 decoy excision	5'-TCAATACATGTAATTTTAGAATTTCAAAGG-3'	
6876 nested bait	5'-GACTGGAGTTCAGACGTGTGCTCTTCCGATCTTTTTCTTACCTCGCACAGTGGC-3'	
Hao1-g1	5'-TCCGTGGATAGAGCTTCCATC-3'	gRNA sequence
Hao1-g2	5'-GTCAACTTCTGTTTTAGGACA-3'	

Appendix Table S2. List of antibodies used in this study

Target	Origin	Type	Label	Method	Supplier	Cat#	Dilution*
α-GO	Rabbit	Polyclonal	N/A	WB/IHC	Home-made		1:5000/1:2000
α-GAPDH	Mouse	Monoclonal	N/A	WB	Sigma-Aldrich	G8795	1:5000
α-GFP	Rabbit	Polyclonal	N/A	WB	Abcam	Ab6556	1:5000
α-rabbit IgG	Donkey	Polyclonal	HRP	WB	GE-Healthcare	NA934V	1:5000
α-mouse IgG	Sheep	Polyclonal	HRP	WB	GE-Healthcare	NA931V	1:5000

*All dilutions were performed in Tris-buffered saline (TBS)-Tween 20 0.05% with non-fat dry milk 5%.
