# Supplementary figures and images for: Efficient and safe therapeutic use of paired Cas9-nickases for primary hyperoxaluria type 1
Source: EMBO Mol Med. 2024 Jan 5;16(1):8. doi: 10.1038/s44321-023-00008-8 (PMC10897483; doi:10.1038/s44321-023-00008-8)

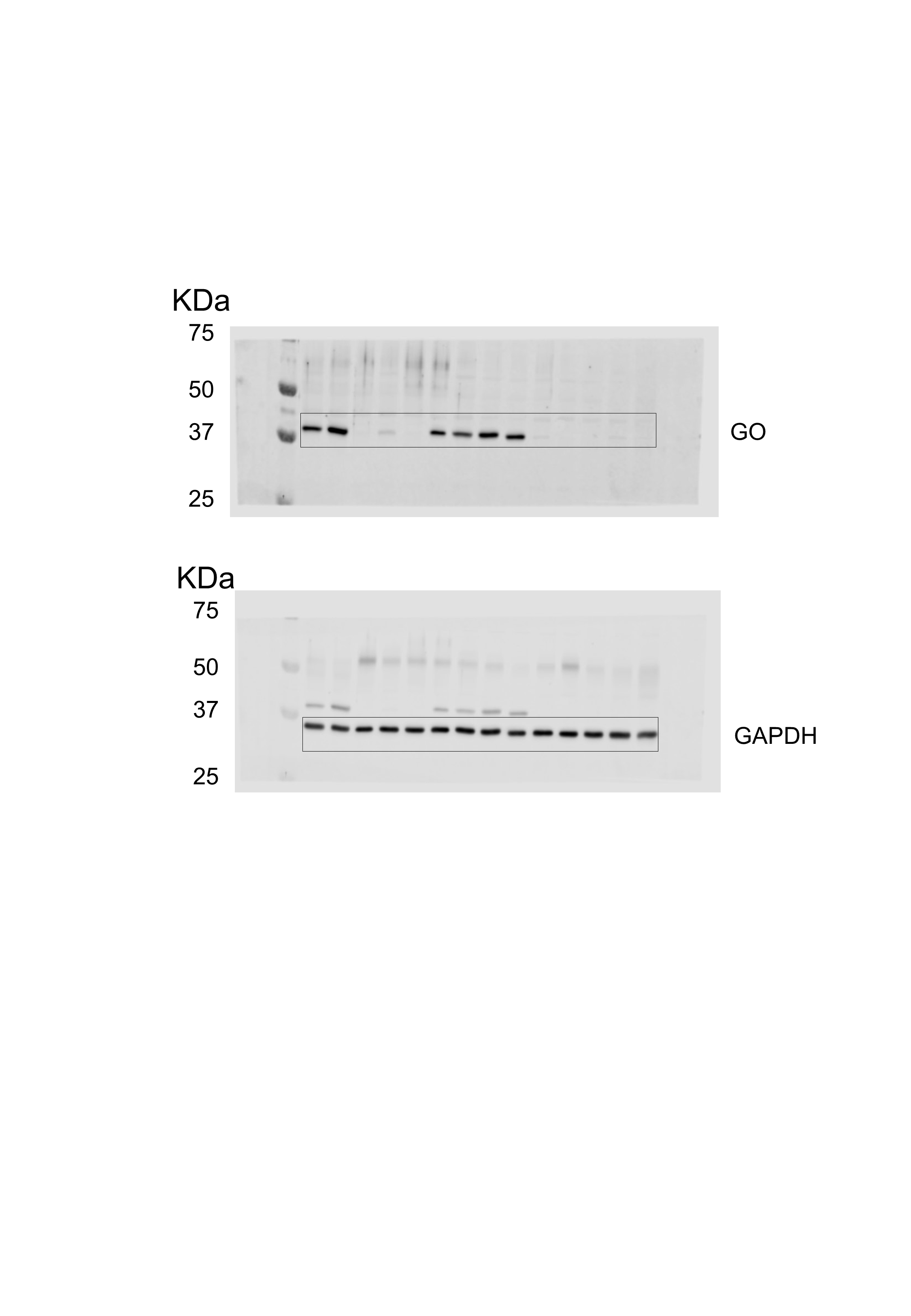

Supplement: Supplementary file 4 — Source Data Fig. 1 [file 44321_2023_8_MOESM4_ESM.zip › Figure1/1C/Figure 1C.tif]

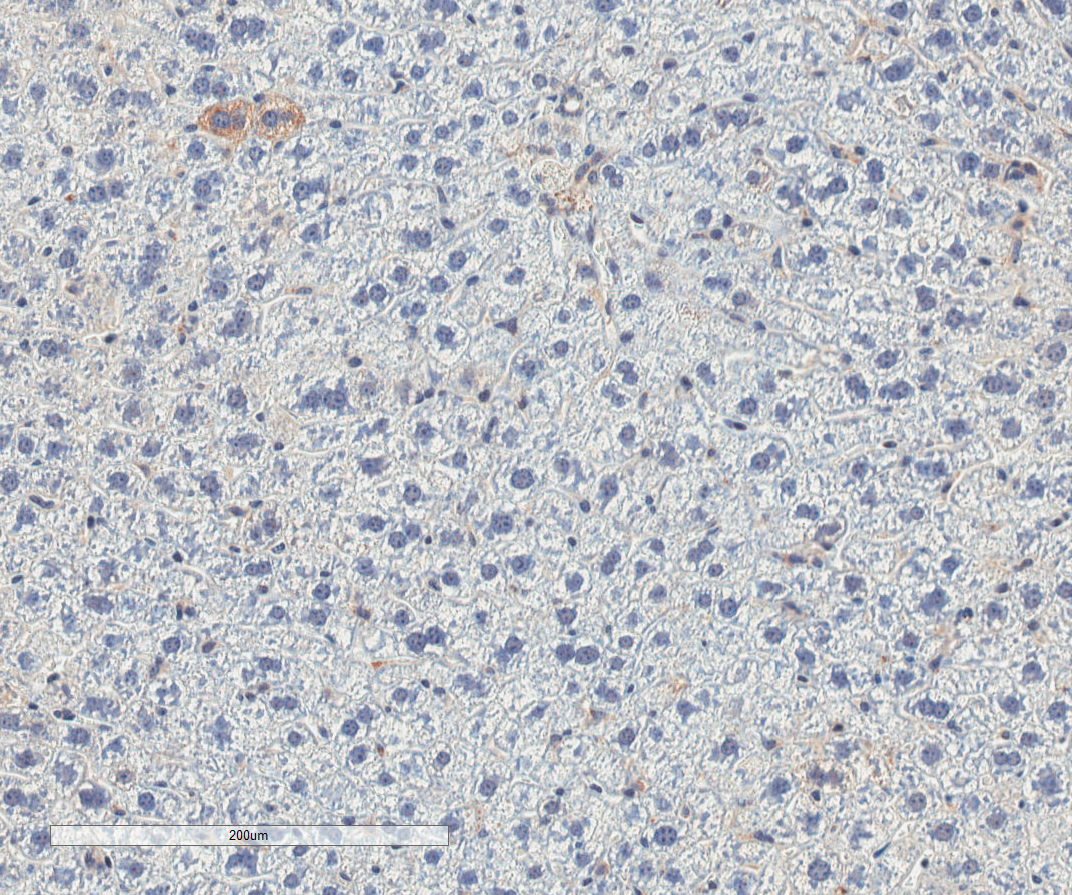

Supplement: Supplementary file 4 — Source Data Fig. 1 [file 44321_2023_8_MOESM4_ESM.zip › Figure1/1D/Figure 1D D10Ag1+g2.tif]

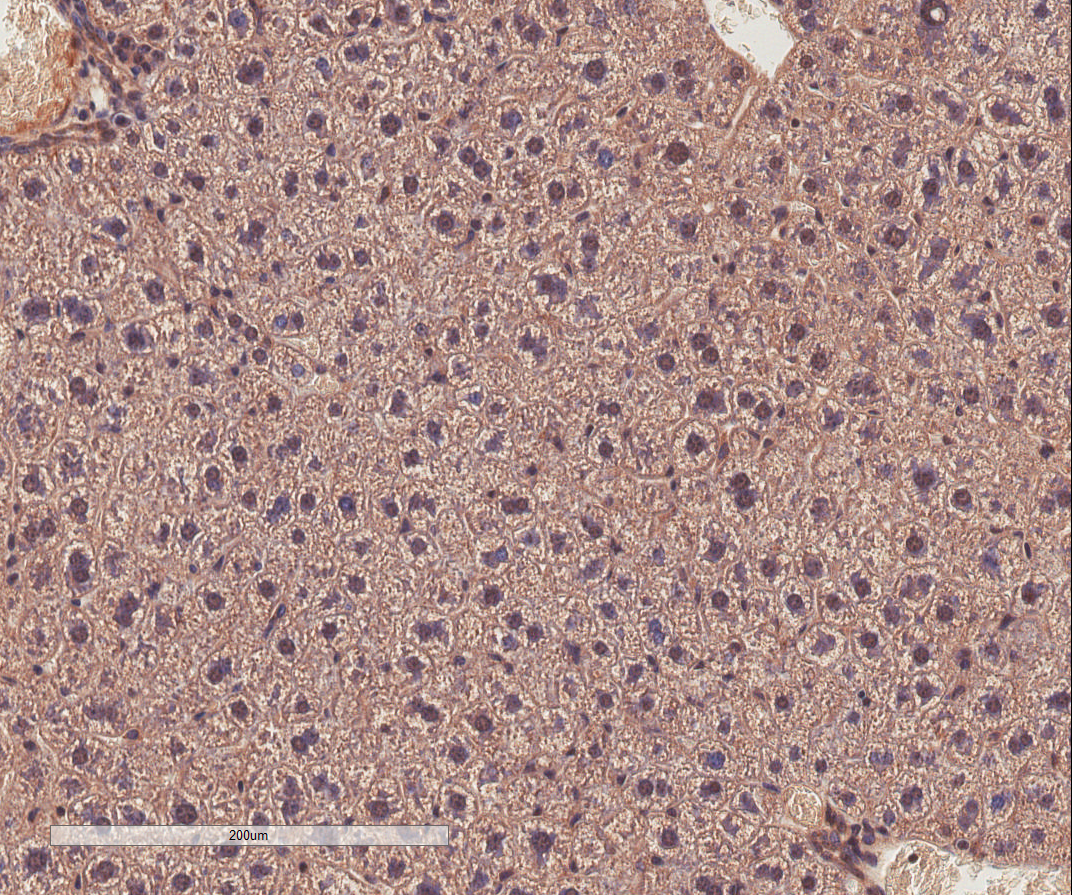

Supplement: Supplementary file 4 — Source Data Fig. 1 [file 44321_2023_8_MOESM4_ESM.zip › Figure1/1D/Figure 1D D10Ag1.tif]

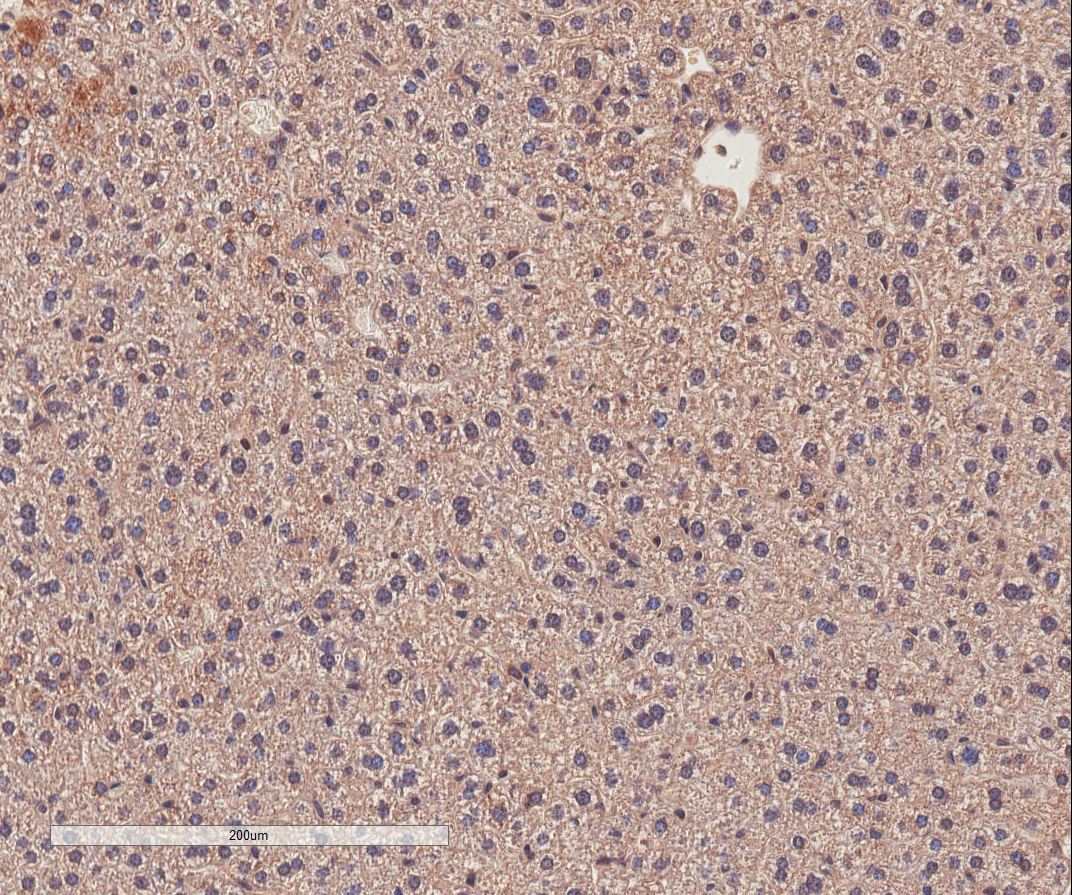

Supplement: Supplementary file 4 — Source Data Fig. 1 [file 44321_2023_8_MOESM4_ESM.zip › Figure1/1D/Figure 1D Saline.tif]

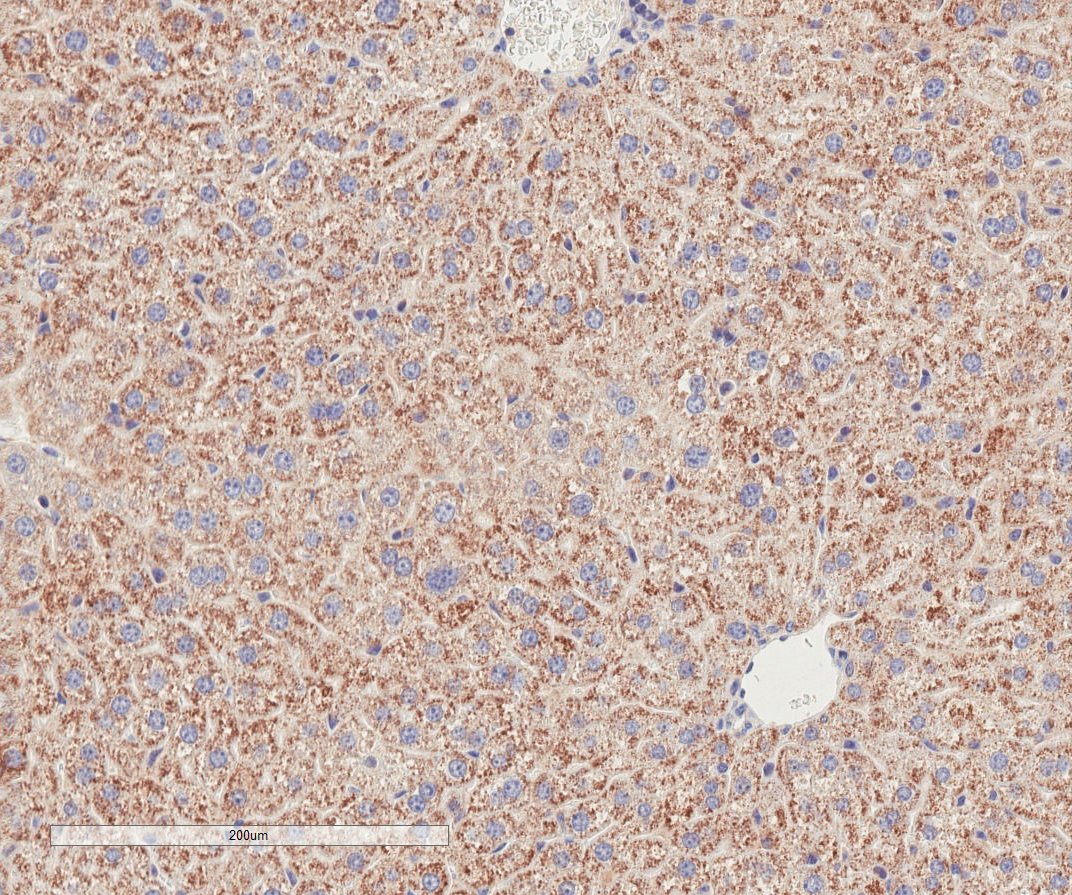

Supplement: Supplementary file 5 — Source Data Fig. 3 [file 44321_2023_8_MOESM5_ESM.zip › Figure3/3B/Figure 3B D10Ag2.tif]

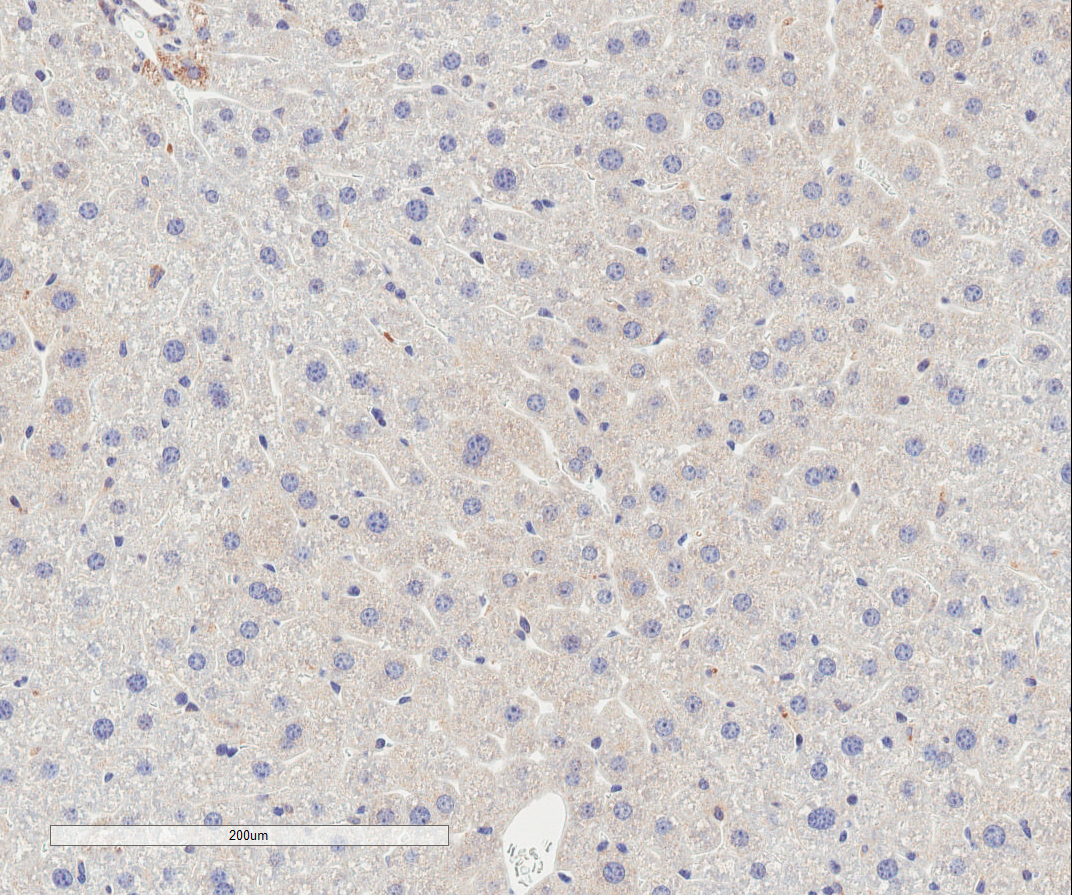

Supplement: Supplementary file 5 — Source Data Fig. 3 [file 44321_2023_8_MOESM5_ESM.zip › Figure3/3B/Figure 3B HD D10Ag1+g2.tif]

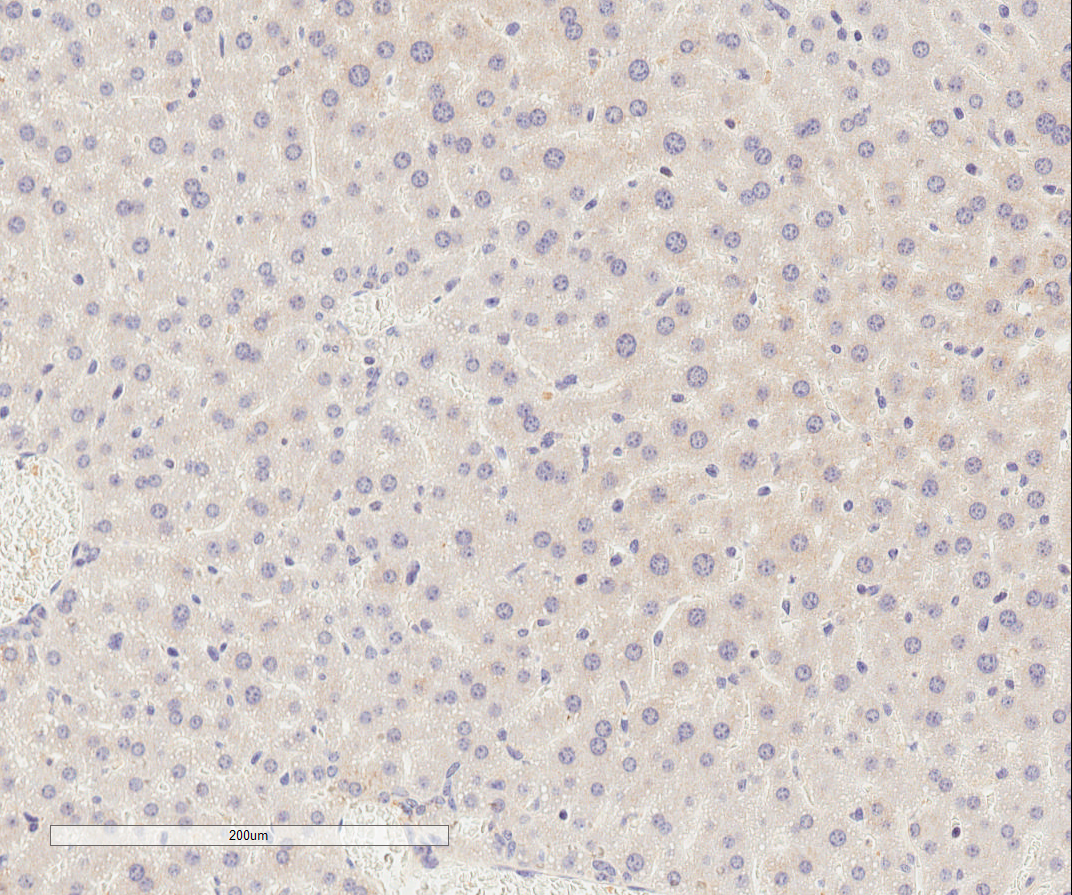

Supplement: Supplementary file 5 — Source Data Fig. 3 [file 44321_2023_8_MOESM5_ESM.zip › Figure3/3B/Figure 3B HD D10Ag1g2.tif]

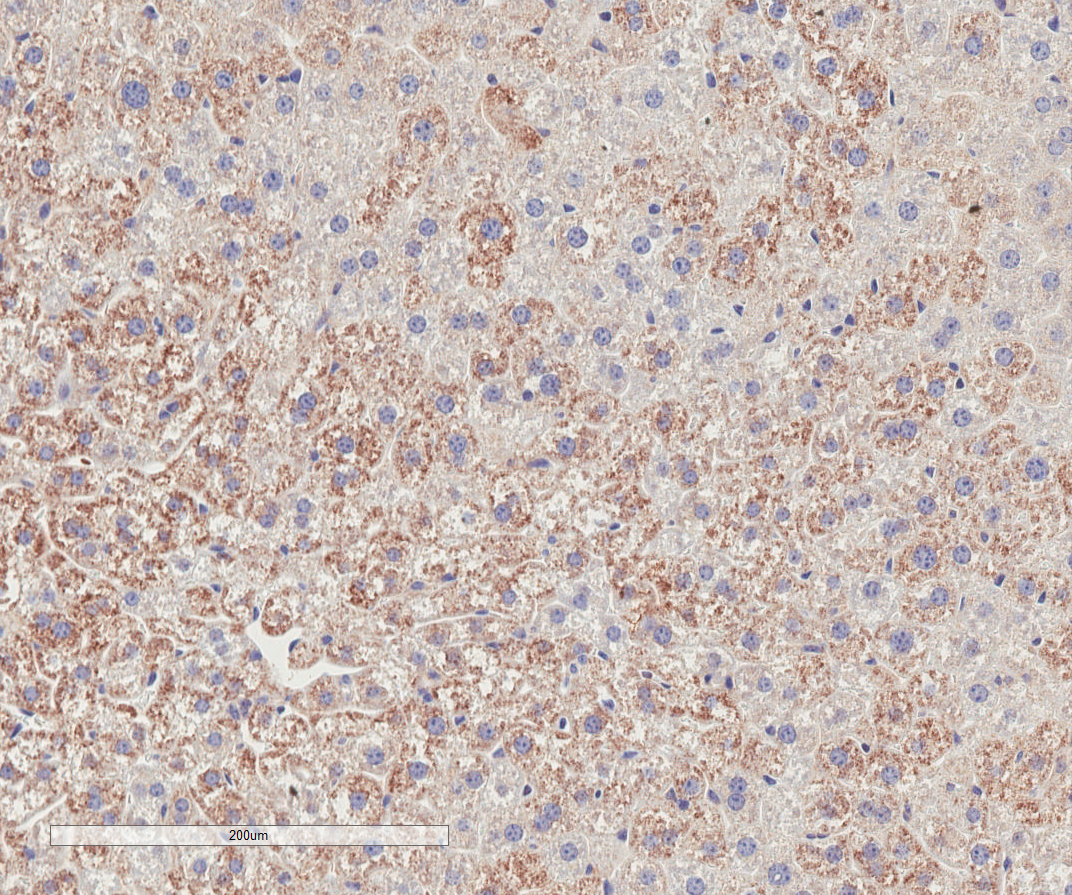

Supplement: Supplementary file 5 — Source Data Fig. 3 [file 44321_2023_8_MOESM5_ESM.zip › Figure3/3B/Figure 3B LD D10Ag1+g2.tif]

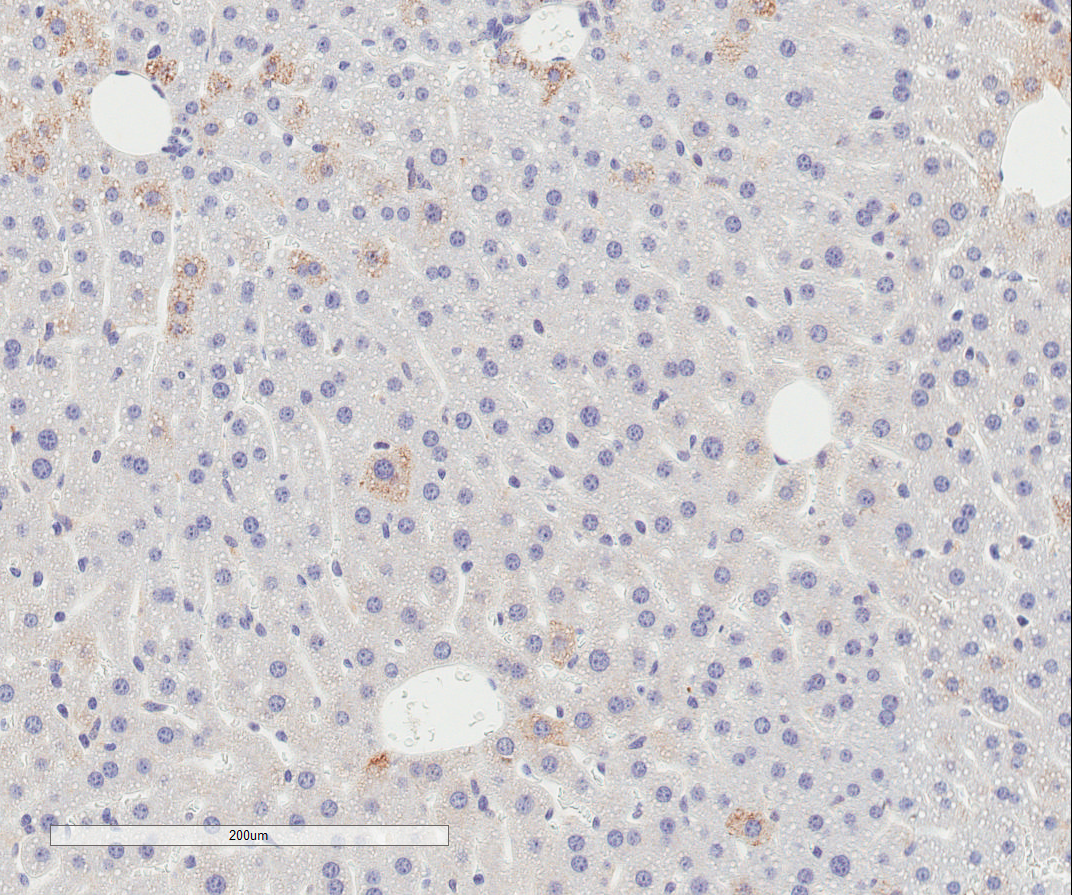

Supplement: Supplementary file 5 — Source Data Fig. 3 [file 44321_2023_8_MOESM5_ESM.zip › Figure3/3B/Figure 3B LD D10Ag1g2.tif]

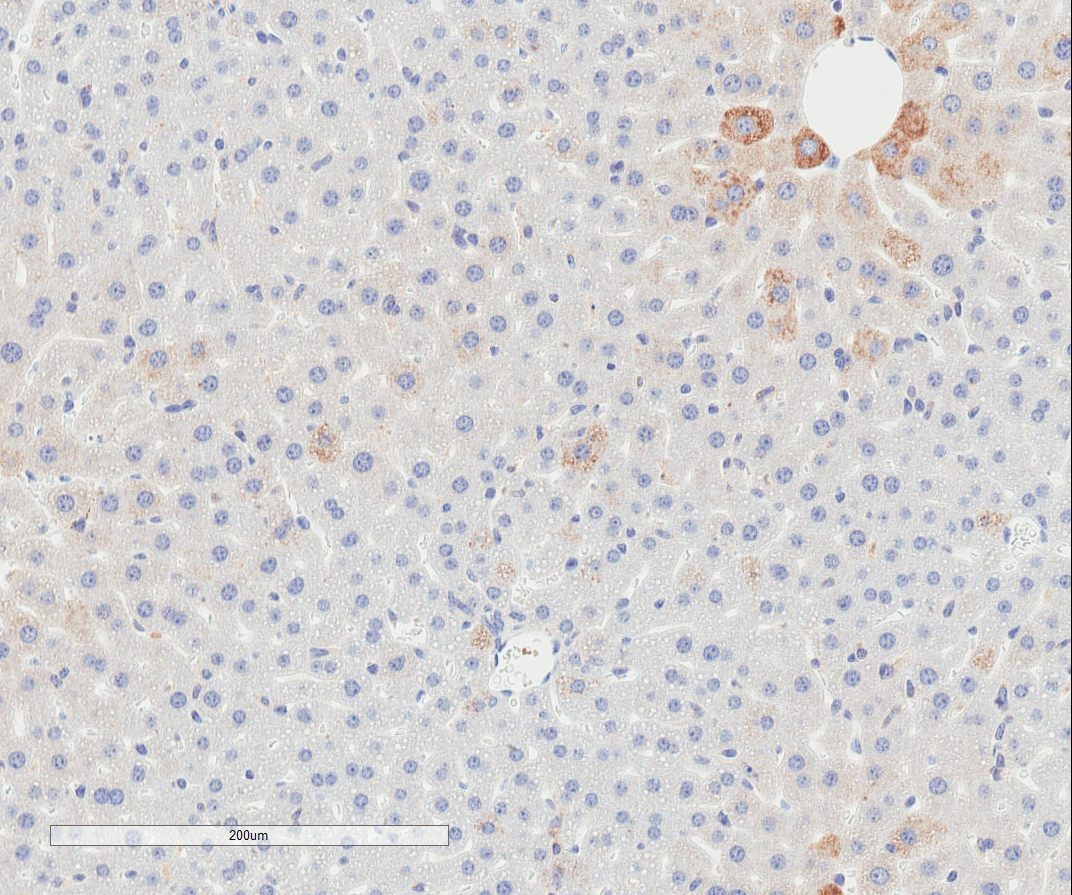

Supplement: Supplementary file 5 — Source Data Fig. 3 [file 44321_2023_8_MOESM5_ESM.zip › Figure3/3B/Figure 3B MD D10Ag1g2.tif]

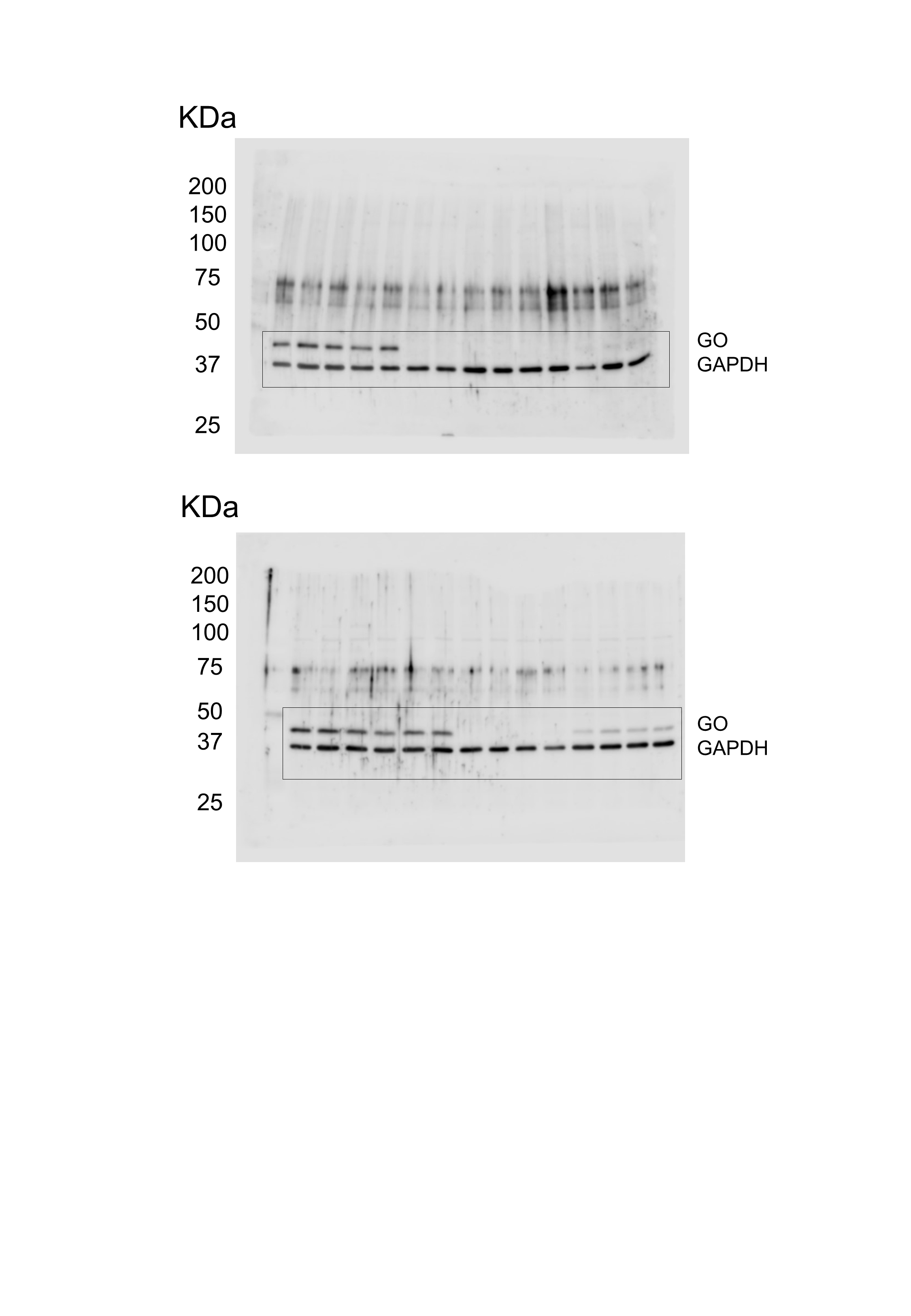

Supplement: Supplementary file 5 — Source Data Fig. 3 [file 44321_2023_8_MOESM5_ESM.zip › Figure3/3C/Figure 3C.tif]
