# Supplementary material for: Efficient and safe therapeutic use of paired Cas9-nickases for primary hyperoxaluria type 1
Source: EMBO Mol Med. 2024 Jan 5;16(1):8. doi: 10.1038/s44321-023-00008-8 (PMC10897483; doi:10.1038/s44321-023-00008-8)
Supplement: Supplementary file 7 — Expanded View Figures [file 44321_2023_8_MOESM7_ESM.pdf]

Expanded View Figures

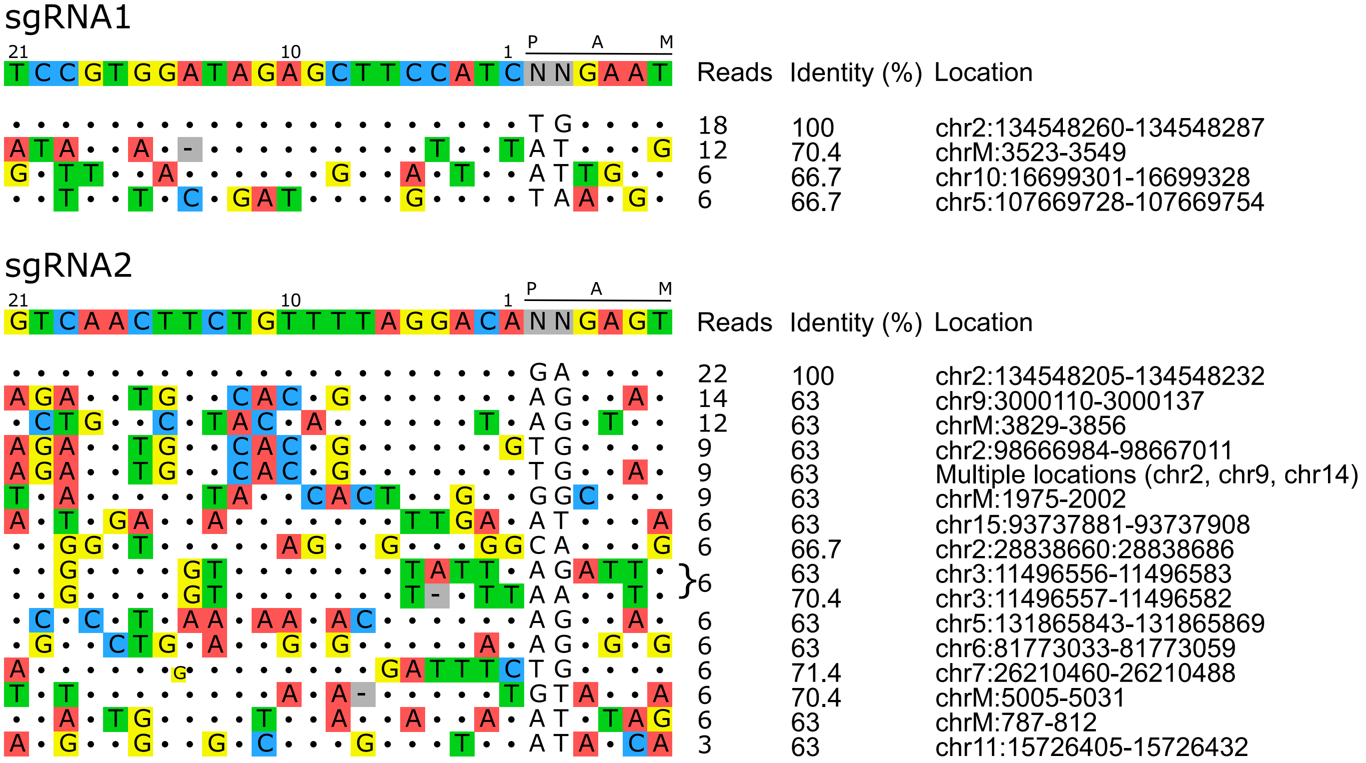

Figure EV1. CIRCLE-seq screening for CRISPR-off-target analysis.

Representation of the off-targets identified by CIRCLE-seq aligned against the intended target site for SaCas9:*Hao1*-g1 and SaCas9:*Hao1*-g2 targeting the *Hao1* gene. The intended target sequence is shown in the top line, the on-target is the first appearing in the below list and the off-target sites are ordered from top to bottom by CIRCLE-seq read count. Perfect base matches are represented as dots while mismatches to the intended target sequence are indicated by coloured nucleotides. Read counts are shown at the end of each line, on the right. Identity of the intended target sequence was calculated and shown at the end of each line, on the right. Chromosomal coordinates for each off-target site are shown at the end of each line, on the right.

© The Author(s)

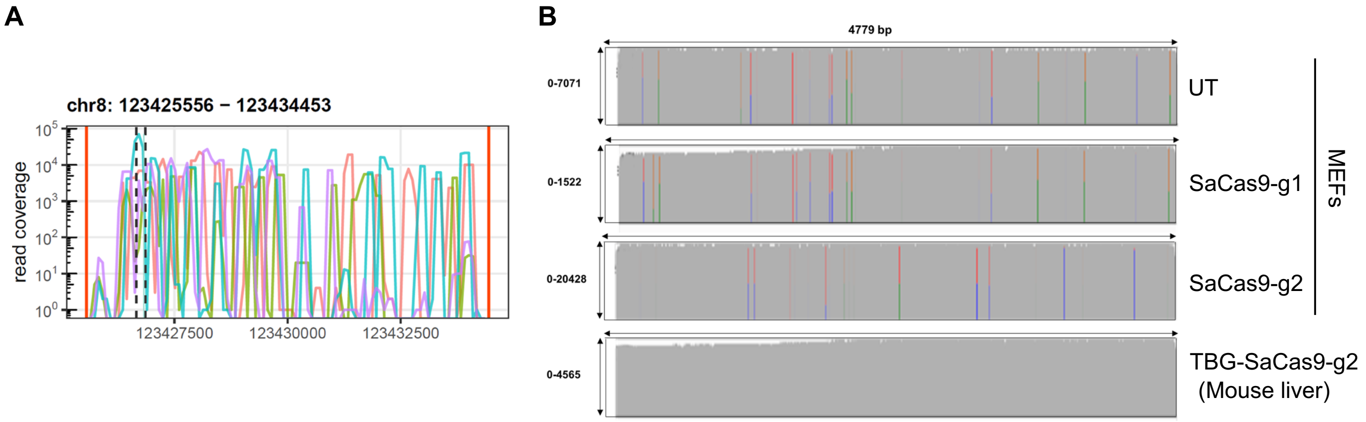

Figure EV2. Targeted long-read sequencing of the OMT detected by CAST-Seq demonstrates the specificity of the selected gRNAs.

(**A**) Coverage plot from the CAST-Seq result showing which reads from chr.8 were translocated onto the on-target site. (**B**) Read coverage of the long-read sequencing spanning 5 kb of chr.8 of untreated MEF (UT-MEF), electroporated MEFs (SaCas9g1-MEF and SaCas9g2-MEF), and a liver sample of PH1 mouse treated with TBG-SaCas9g2. $N = 1$ biological replicate per condition is shown. The deletion read coverage was normalized to the total amount of reads covering each position of the amplicon (mean ± SD).

 © The Author(s)
